# Antifungal effects of coating chitosan particles on polymethylmethacrylate acrylic resin: An in vitro study

**DOI:** 10.1186/s12903-026-09845-8

**Published:** 2026-09-11

**Authors:** Mehwish Shoro, Muhammad Rizwan Memon, Aamir Mahmood Butt, Hina Memon, Osama Khattak, Gotam Das, Waled Abdulmalek Alanesi

**Affiliations:** 1 Department of Prosthodontics, Institute of Dentistry, Liaquat University of Medical & Health Sciences, Jamshoro, Pakistan; 2 Department of Prosthetic Dental Sciences, College of Dentistry, Jouf University, Sakaka, Kingdom of Saudi Arabia; 3 Department of Restorative Dentistry, College of Dentistry, Jouf University, Sakaka, Saudi Arabia; 4 Department of Prosthodontics, College of Dentistry, King Khalid University, 61421 Abha, Kingdom of Saudi Arabia; 5 Restorative and Prosthodontic Department, College of Dentistry, University of Science and Technology, Sana’a, Yemen

**Keywords:** Fungal infections, Poly methyl methacrylate, Chitosan particles, Denture base resin

## Abstract

**Background:**

Poly methyl methacrylate (PMMA) acrylic resin is widely used for denture fabrication due to its biocompatibility and low cost; however, its porous surface makes it highly susceptible to colonization by Candida albicans. Biofilm formation by this organism poses a persistent clinical challenge due to its resistance to antifungal treatments. Chitosan (CS), a naturally derived biopolymer with proven antimicrobial properties, represents a promising solution. This study evaluated the in vitro antifungal effects and growth inhibition of a novel chitosan nano particle coating on Candida albicans applied to heat-cured acrylic resin.

**Materials and methods:**

A total of sixty standard-dimension, freshly fabricated, and sterilized polymethylmethacrylate plates were randomly allocated into two groups. Thirty in the control group were treated with uncoated acrylic resin denture base plates, and thirty in the study group were treated with acrylic resin denture base coated with chitosan nanoparticles. All the specimens were sterilized by autoclaving at 15 psi/121°C for 15 min. The diameter of the zone of growth inhibition was assessed clinically with the naked eye and measured around each specimen in mm using a measuring scale. All data were collected on a pro forma.

**Results:**

Mean inhibiting fungal growth was 1.6958 ± 0.95 mm in the study group and 0.2783 ± 0.74 mm in the control group. Significant mean difference was observed between groups (difference = 1.41743; *p* < 0.001). The results of the current study may show a decrease in antifungal activities.

**Conclusion:**

We conclude that chitosan in solutions is more effective at reducing the viability of C. albicans biofilm on poly-methyl methacrylate resin than not using chitosan. Taking the above into consideration, the studied concept of modifying denture base materials with chitosan requires improvement.

**Supplementary Information:**

The online version contains supplementary material available at https://doi.org/10.1186/s12903-026-09845-8.

## Background

Candida albicans is a commensal yeast that normally resides on human mucosal surfaces, such as the oral cavity, gastrointestinal tract, vagina, and skin [1]. It is part of the normal microbiota in healthy individuals but can become opportunistic when the host’s immune system is compromised or the normal microbial balance is disturbed. Candida can cling to and spread across various surfaces. For example, biomaterials such as polymethyl methacrylate, an acrylic denture base material. Due to its stability in the oral cavity, low cost, light weight, ease of processing, and attractive qualities, it is widely used as a denture base material globally [2]. A typical inflammatory response associated with wearing dentures, denture-induced stomatitis, is typically caused by Candida species. Less prevalent causes of denture stomatitis include allergic contact reactions and mechanical trauma [3].

Denture stomatitis, periodontitis, gingivitis, caries, and peri-implantitis are all correlated with oral biofilm. It is difficult to control its formation in the oral cavity [4, 5]. Among Candida species, the most common isolate in oral mucosal infections, such as denture stomatitis (DS), is Candida albicans. With a prevalence of up to 70%, DS is a common mucosal disorder among denture wearers, characterized by inflammation, erythema, and/or hyperplasia of the oral mucosa. It is complex and linked to denture biofilms, inadequate cleaning, subpar denture material, and the use of dentures at night [6, 7].

The main structural element of crustacean and arthropod shells is chitin [2, 3]. Chitosan, a polysaccharide made up of units of glucosamine and N-acetylene glucose amine connected by beta (1–4) bonds, is produced when chitin is partially deacetylated [7]. Chitosan has been used in several biomedical applications because of its antimicrobial activity, low toxicity, biodegradability, and biocompatibility. Chitosan has demonstrated antifungal properties, especially against Candida albicans [3, 8].

In one study, fungal cells were killed, and fungal biofilm on dental polymethyl methacrylate was inhibited using the quaternary ammonium chitosan multilayering technique [9]. An alternative that shows promise for treating and preventing denture stomatitis is the combination of chitosan and tissue conditioners. The antifungal properties of the chitosan-containing tissue conditioner composite were reported in an additional study and an experimental formulation, providing evidence of this [10].

While conventional treatments for denture stomatitis rely on antifungal medications and chemical disinfectants like chlorhexidine, these strategies possess significant drawbacks. The global rise in antifungal drug resistance, alongside issues such as chlorhexidine-induced acrylic staining and increasing patient allergies, necessitates the development of alternative antifungal strategies [9]. Chitosan, a naturally derived biopolymer, offers a promising solution due to its proven antimicrobial properties and lack of conventional resistance mechanisms [11]. This study introduces a novel approach by evaluating the direct surface coating of PMMA with synthesized chitosan nanoparticles—a simple, cost-effective modification intended to inhibit fungal growth without compromising the bulk properties of the resin.

The study aimed to apply diluted chitosan nanoparticles to the most widely used denture base material, PMMA. The study showed a decrease in antifungal activity; ultimately, the quality of life of denture wearers will improve, and they will be able to tolerate the prosthesis better, as denture stomatitis has been a major cause of prosthesis withdrawal. This study will have a tremendous positive impact on society by introducing a cost-effective material for making dental prostheses. To compare the mean reduction in antifungal activity of poly methyl methacrylate denture base resin, coated with chitosan nanoparticles, with uncoated denture base resin. This study aims to test the hypothesis that PMMA acrylic resin coated with chitosan and untreated PMMA acrylic resin have similar antifungal activities against Candida albicans.

## Materials and methods

The study was an experimental study (in vitro). A denture base acrylic resin specimen was fabricated at the Prosthodontics Laboratory of the Institute of Dentistry, Liaquat University of Medical & Health Sciences (LUMHS), Jamshoro, Sindh, Pakistan and antifungal activity was tested at the Medical Research Centre, LUMHS Jamshoro.

### Mould and specimen preparation

**Fig. 1:**
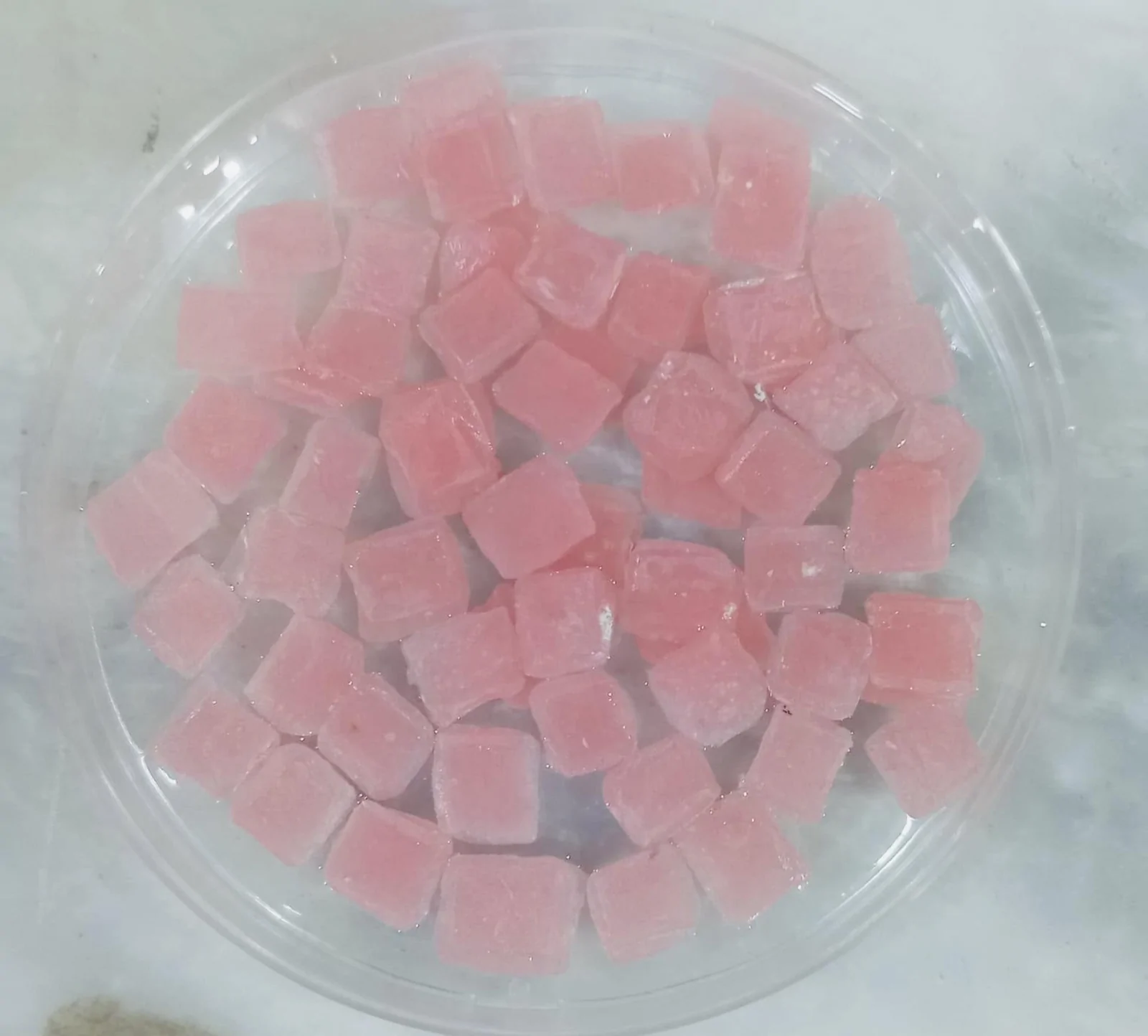
Standardized test specimens (6 × 6 × 2 mm) fabricated from heat-cured PMMA acrylic resin and stored in artificial saliva to simulate oral environmental conditions prior to chitosan application

The 60 specimens were fabricated, with dimensions of 6 × 6 × 2 mm, using a single brand of Vertax (Soesterberg, Netherlands) heat-cure acrylic resin denture base material. Specimens were fabricated by cutting hard elastic foil of 2 mm thickness into plastic specimens (6 × 6 × 2 mm). Flasking was accomplished by the conventional method, followed by packing, and then curing was performed by placing the clamped flask in a thermostatically controlled water bath for 30 min at 70 °C, then at 100 C for another 30 min. The flask was allowed to cool on the bench for 30 min after the curing process was completed. De-flasking was done to remove acrylic specimens from their stone models (Di-Stone Dent USA). Any flashes were removed, and finishing was performed using an acrylic bur (Fig. 1). All the specimens were sterilized by autoclave at 15 pounds/inch^2^/121 °C for 15 min [8] Randomization was done through a lottery system. The control group consists of uncoated acrylic resin denture base plates, with 30 specimens. The study group consists of acrylic resin denture bases coated with chitosan particles, with 30 specimens. Initially, a group of specimens was stored in artificial saliva (Wet Mouth, ICPA) at 37 °C ± 1 °C for 7 days.

A medium-molecular-weight chitosan solution (Sigma-Aldrich, St. Louis, MO, USA) was prepared with a degree of deacetylation of 75% or higher. For four to six hours at room temperature, a clear and uniform solution was produced by dissolving chitosan powder in a 0.1% (v/v) acetic acid solution with continuous magnetic stirring. According to reports, chitosan at 1% (w/v) exhibits effective antibacterial activity while still forming a sufficient film. To achieve consistency and remove any undissolved particles, the solution was filtered through Whatman filter paper. To maximize antifungal activity and improve biocompatibility, the solution’s pH was adjusted to around 5.5-6.0 using 1 N sodium hydroxide (NaOH). Next, the mixture was sonicated for 20 min to enhance chitosan nanoparticle dispersion and reduce particle aggregation. To remove surface impurities and improve coating adherence, all PMMA specimens were ultrasonically cleaned in distilled water for 10 min before being air-dried under sterile circumstances.

**Fig. 2:**
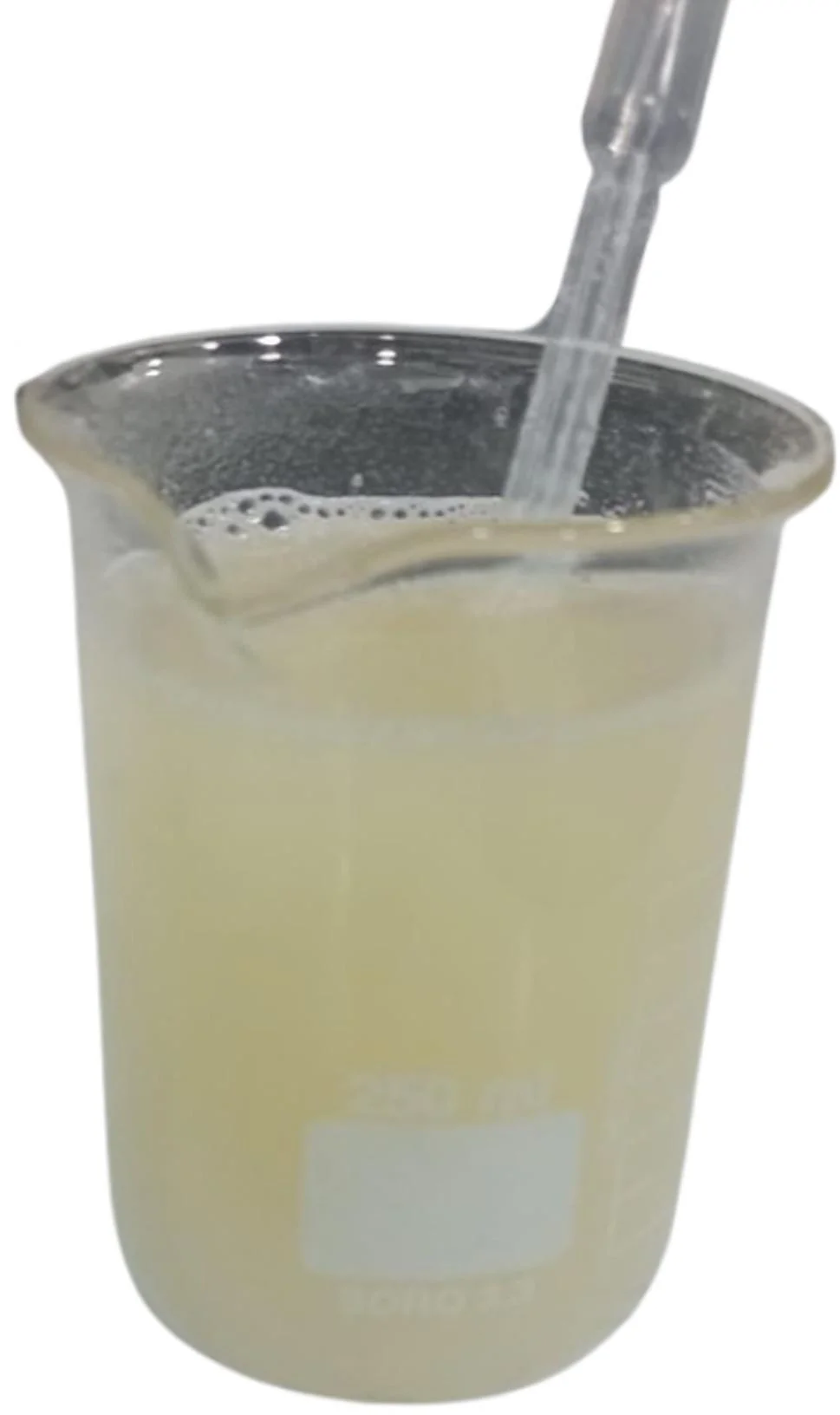
Preparation of the chitosan nanoparticle suspension, achieved by dissolving medium-molecular-weight chitosan in a 0.1% acetic acid solution under constant magnetic stirring and subsequent ultrasonication to ensure optimal nanoparticle dispersion

Thirty acrylic resin specimens were coated with chitosan nanoparticles diluted in 0.1% acetic acid. The coating was applied uniformly with a camel-hair brush in a single direction to ensure consistent surface coverage. While the immediate application was standardized, the long-term stability, release kinetics, and physical uniformity of the coating layer were not quantitatively characterized in this initial in vitro model. Candidal suspension was prepared using a standardized cell concentration of approximately 6 × 10⁶ CFU/mL, equivalent to McFarland standard tube No. 2. The McFarland standard solution (tube No. 2) was prepared by mixing 0.2 mL of 1% barium chloride with 9.8 mL of 1% sulfuric acid (H₂SO₄). The Candida albicans strain used in this study was obtained from the Microbiology Laboratory culture collection. A loopful of Candida albicans colonies was suspended in sterile distilled water and mixed thoroughly until the turbidity matched that of McFarland standard tube No. 2, as confirmed using a UV spectrophotometer (Columbus, OH, USA) at a wavelength of 600 nm (OD₆₀₀), to ensure standardization of cell density (Fig. 2). After the formation of the candidal cell suspension, antifungal activity was tested through the Disk Diffusion Method.

## Disk diffusion method (Sensitivity test)

**Fig. 3:**
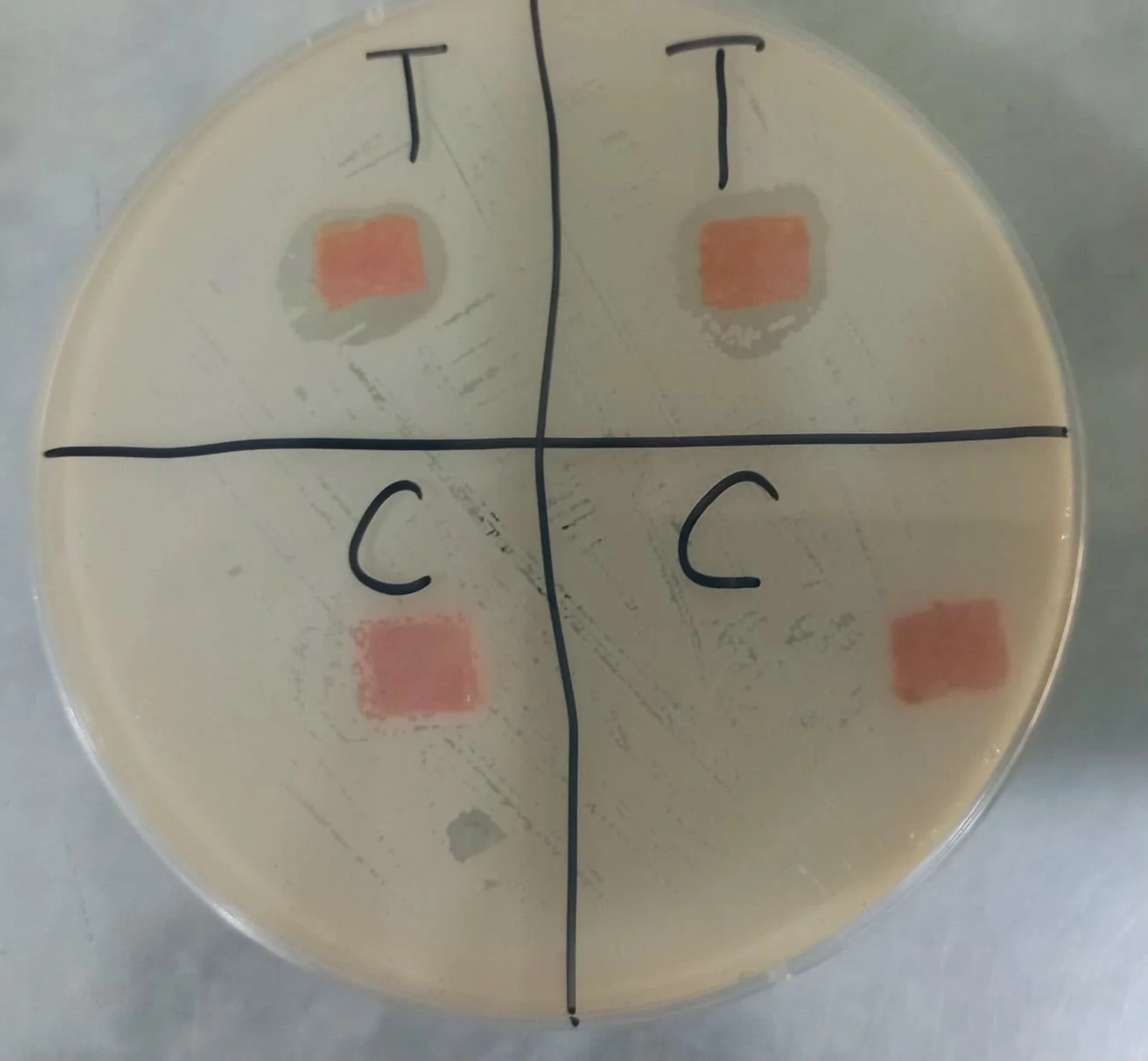
Disk diffusion assay demonstrating the formation of a distinct zone of growth inhibition around the chitosan-coated test samples (T) compared to the uncoated control samples (C), indicating the diffusion and antifungal efficacy of the chitosan against Candida albicans

Candidal cell suspension was streaked on Sabouraud Dextrose agar using an L-shaped spreader. After 15 min, acrylic resin specimens from both groups were added to the agar surface using sterile forceps, and the plates were incubated at 37 °C for 24 h. The diameter of the zone of growth inhibition was assessed clinically with the naked eye and measured around each specimen in mm using a measuring scale (Fig. 3). A single operator performed the entire procedure.

## Results

A total of sixty specimens of standard dimensions, freshly fabricated and sterilized. Acrylic plates were randomly allocated into two groups. Thirty in the control group were treated with uncoated acrylic resin denture base plates, and thirty in the study group were treated with acrylic resin denture base coated with chitosan particles. Comparison of the mean reduction in antifungal activity of poly methyl methacrylate denture base resin, coated with chitosan nanoparticles, with uncoated denture base resin is reported in Table 1. Mean inhibiting fungal growth was 1.6958 ± 0.95 mm in the study group and 0.2783 ± 0.74 mm. Signi in the control group significant mean difference was observed between groups (difference = 1.41743; *p* < 0.001). Table 2 shows the t-test group statistics. In our data, Levene’s test shows a non-significant P-value, so we can proceed with the tests under the assumption of equal variances and conclude that we do not have sufficient evidence to reject the null hypothesis. However, given the significant p-value of *p* < 0.001, which is lower than 0.05, we reject the null hypothesis and accept that there is a significant difference in the mean values of the two groups.

**Table 1:** Statistical comparison of mean antifungal activity zones between control and test groups, showing the relative antifungal efficacy of each formulation

Group Statistics					
Zone of inhibition in millimetres	Group	N	Mean	Std. Deviation	Std. Error Mean
	Test	30	1.6958	0.95905	0.17510
	Control	30	0.2783	0.74619	0.13624

**Table 2:** Descriptive statistics (mean, standard deviation, and sample size) for each group included in the T-test comparing antifungal activity

		Levene’s test for equality of variances		T- Test for Equality of Means 95% confidence interval of difference						
		F	sig	t	df	Sig. (2 tailed)	Mean diff	St. error diff	lower	upper
Zone of inhibition in millimetres	Equal variances assumed	5.584	0.21	6.389	58	0.000	1.41743	0.22185	0.97334	1.86152
	Equal variances not assumed			6.389	54.695	0.000	1.41743	0.22185	0.97277	1.86209

## Discussion

The current recommendation for treating denture stomatitis calls for antifungal medication in addition to denture disinfection [10, 11]. Nonetheless, the global prevalence of antifungal drug resistance has increased, and the regular use of antifungal medications may contribute to its emergence through the overexpression of drug efflux pumps or mutations in target enzymes [12]. Patients with resistant infections require alternative antifungal agents that target distinct fungal pathways. The mechanisms by which chitosan is thought to work, such as interfering with the function of cell membranes and with DNA and RNA synthesis, differ from those of medications currently on the market. Chitosan may therefore be helpful for patients who are not responding to their present antifungal medicines [13]. The observed zone of inhibition primarily reflects the antifungal growth inhibition caused by the diffusion of chitosan nanoparticles from the PMMA surface into the surrounding agar medium. While this demonstrates potent fungistatic or fungicidal activity, it does not directly measure true antibiofilm properties, such as the disruption of extracellular polymeric substances or established biofilm architectures. These results align with broader efforts to develop novel antifungal surfaces; for instance, recent advancements in antimicrobial peptide (AMP)-based strategies against Candida species highlight the growing need for materials that can disrupt fungal cell membranes without inducing resistance [13–15]. Antimicrobial peptides (AMPs) are an emerging group of surface modifying agents for use in conjunction with oral biomaterials, such as implants. The disruptive nature of AMPs toward microbial cell membranes, prevention of biofilm development, and lack of resistance to common antimicrobial compounds make AMPs highly desirable over traditional chemical disinfectants and antifungal drugs. Studies on AMP-coated implant surfaces have demonstrated the capability of AMPs to effectively inhibit the growth of many oral pathogenic bacteria (such as Streptococcus spp.) as well as peri-implant biofilm communities [14], demonstrating the potential for AMPs to be incorporated into prosthetic materials. In addition to their bactericidal properties, there are multiple examples of AMPs that have been identified as having potent antifungal capabilities. For example, magainin and other derivatives of magainin have shown broad spectrum fungicidal activity against various strains of Candida species by causing disruption of the fungal cell membrane and/or intracellular targeting [16, 17]. Another area of interest includes marine-derived AMPs which have demonstrated excellent biocompatibility along with effective inhibition of both fungal and bacterial biofilms [18]. Therefore, incorporation of AMP based coatings onto PMMA or combination of AMPs with chitosan-based coating systems could be a viable long-term solution for creation of multi-functionality within denture base materials to provide sustained antifungal protection from complex polymicrobial biofilms.

In this study, there was a significant difference in mean reduction in antifungal activity between the groups. the poly methyl methacrylate denture base resin coated with chitosan nanoparticles versus the uncoated denture base resin. A study that used different forms of chitosan for anti-candidal activity, such as 0.1% and 0.2% acetic acid and 3 and 6 mg/ml of chitosan oligomer, also reported similar results. After a 12-hour immersion, the mean relative viability was 6.60 + 4.75% and 12.72+_6.96% for 3 and 6 mg/ml oligomer and 11.68+_4.81% and 18.08+_6.20% for 3 and 6 mg/ml 30 kDa chitosan, respectively, in comparison to the control [10]. In a different study, fungal cells were killed and fungal biofilm on dental polymethyl methacrylate was inhibited by the quarternary ammonium chitosan multilayering technique [9].

Additionally, low-molecular-weight chitosan (107 kDa) nanoparticles demonstrated good activity against C. albicans biofilms with negligible adverse effects on PMMA, according to a recent study [19]. Although the chitosan utilized in this study had a higher molecular weight than that used previously, its anti-candida activity might be enhanced by its preparation into nanoparticles. However, compared to the nanoparticle formulation, the chitos [20]. Denture stomatitis (DS) can be treated with CS coatings, CS mouthwash, or CS solution, among other forms of CS, which have been demonstrated to have antifungal and antibiofilm properties. The strategies outlined yielded encouraging outcomes and validated CS’s antifungal and antibiofilm properties [21–24].

In 2019, Walczak et al. [25] conducted a study to modify PMMA with CS-salt and characterize its antifungal and surface properties in vitro, thereby creating a novel antifungal denture base material. For this reason, the antifungal properties of CS hydrochloride or CS-glutamate salt solutions in different concentrations were found. The specimens were subjected to a C. albicans biofilm assay to assess the antifungal qualities of the resins modified with CS salt. Contact profilometry was also used to measure the roughness of the specimens. They concluded that, before analyzing the antifungal properties of PMMA resins modified with CS salts, each CS salt’s antifungal activity was first assessed. According to the relative growth analysis, the antifungal properties of both CS salts were good. CS salts significantly reduced relative fungal growth in all groups compared with the control group. Although the CS salts exhibited good antifungal activity, their effectiveness is diminished when combined with denture base material [26, 27]. Previous research on the antimicrobial and antibiofilm properties of CS produced encouraging findings [28–29].

In one of the recent studies, various types of chitosan were tested for anti-candidal activity, including 0.1% and 0.2% acetic acid and 3 and 6 mg/ml of chitosan oligomer. The mean relative viability compared to the control after 12 h of immersion was 6.60 ± 4.75% and 12.72 ± 6.96% for the 3 and 6 mg/ml oligomer, respectively, and 11.68 ± 4.81% and 18.08 ± 6.20% for the 3 and 6 mg/ml 30 kDa chitosan, respectively [30]. Furthermore, we found that at comparable concentrations, most chitosan solutions exhibited more potent antifungal activity than acetic acid alone. Therefore, it is possible that acetic acid and chitosan both influenced the fungicidal properties of chitosan solutions. Since acetic acid and chitosan are both very affordable and biocompatible (at low concentrations), we propose that chitosan solutions could be used as a low-risk and safe denture cleaning solution. However, more research is required to determine the detrimental impact of chitosan on the characteristics of acrylic resin [31].

Chlorhexidine exerts an antimicrobial effect by binding to the negative charges on microbial cell walls, causing cell components to leak [32–33]. Chlorhexidine has been suggested as a denture cleaning solution; however, frequent use may cause acrylic resins to become stained [34–35]. Furthermore, patients might be allergic to chlorhexidine, a condition that is becoming more and more common and can cause a wide range of symptoms, from minor reactions to fatal ones [35]. It is commonly known that when microorganisms, such as Candida albicans, are grown in biofilm, their resistance to antimicrobial agents increases [34].

This study has several limitations. First, the antifungal efficacy was evaluated using a single parameter (the zone of inhibition), which reflects growth inhibition rather than direct antibiofilm activity. Second, the coating efficiency, uniformity, and long-term stability of the chitosan nanoparticles on the PMMA surface were not physically or chemically characterized. Future studies must incorporate biofilm-specific assays (e.g., XTT reduction, confocal microscopy) and material characterization techniques (e.g., scanning electron microscopy) to validate the durability and clinical reliability of this coating strategy.

## Conclusion

The antifungal efficacy of PMMA denture base resin against Candida albicans is significantly enhanced by the incorporation of a chitosan nanoparticle coating. This approach could prevent denture-induced stomatitis.

## Supplementary Information

Supplementary Material 1.

## Data Availability

The datasets used and/or analysed during the current study are available from the corresponding author on reasonable request.

## Abbreviations

PMMA: Poly methyl methacrylate
CS: Chitosan
DS: denture stomatitis
UV: Ultraviolet-Visible
AMP: Antimicrobial peptide
